# The diversity of uncharacterized antibiotic resistance genes can be predicted from known gene variants—but not always

**DOI:** 10.1186/s40168-018-0508-2

**Published:** 2018-07-07

**Authors:** Johan Bengtsson-Palme

**Affiliations:** 1 Wisconsin Institute for Discovery, University of Wisconsin-Madison, 330 North Orchard Street, Madison, WI 53715 USA; 2 Centre for Antibiotic Resistance research (CARe) at University of Gothenburg, Gothenburg, Sweden; 3 Department of Infectious Diseases, Institute of Biomedicine, The Sahlgrenska Academy, University of Gothenburg, Guldhedsgatan 10, SE-413 46 Gothenburg, Sweden

**Keywords:** Antibiotic resistance, Diversity measurements, Gene diversity, Risk ranking, Microbial biodiversity

## Abstract

**Background:**

Antibiotic resistance is considered one of the most urgent threats to modern healthcare, and the role of the environment in resistance development is increasingly recognized. It is often assumed that the abundance and diversity of known resistance genes are representative also for the non-characterized fraction of the resistome in a given environment, but this assumption has not been verified. In this study, this hypothesis is tested, using the resistance gene profiles of 1109 metagenomes from various environments.

**Results:**

This study shows that the diversity and abundance of known antibiotic resistance genes can generally predict the diversity and abundance of undescribed resistance genes. However, the extent of this predictability is dependent on the type of environment investigated. Furthermore, it is shown that carefully selected small sets of resistance genes can describe total resistance gene diversity remarkably well.

**Conclusions:**

The results of this study suggest that knowledge gained from large-scale quantifications of known resistance genes can be utilized as a proxy for unknown resistance factors. This is important for current and proposed monitoring efforts for environmental antibiotic resistance and has implications for the design of risk ranking strategies and the choices of measures and methods for describing resistance gene abundance and diversity in the environment.

**Electronic supplementary material:**

The online version of this article (10.1186/s40168-018-0508-2) contains supplementary material, which is available to authorized users.

## Background

The looming antibiotic resistance crisis is recognized by the WHO as one of the most urgent threats to modern healthcare [1]. Although resistance is overwhelmingly a clinical problem, much evidence points towards an environmental origin of many resistance genes [2–5]. Given the enormous genetic diversity of environmental bacteria, this should not be a surprise. Antibiotics are naturally occurring in microbial communities [2], and some resistance genes could have evolved as a defense system to antimicrobial molecules secreted by other microbes [6]. Yet other genes may not at all have had a resistance function in natural settings, but only confer resistance when overexpressed or in the face of anthropogenic antibiotic selection. Together, these genes constitute the environmental *resistome*, a term encompassing several types of genes, including known clinical resistance genes, genes closely homologous to known resistance genes which likely confer the same resistance phenotypes, genes already confirmed to have resistance functions as detected by functional metagenomics screens but which do not share homology to known resistance genes, as well as currently completely unknown resistance genes for which we know neither the sequence nor the function [7, 8].

The recognition that the environment could serve as a source for resistance genes to human pathogens has spurred interest in investigating the distribution of resistance genes in various environments to better understand this process [9–14]. Large-scale quantification efforts of resistance, regardless if they rely on PCR-based methods or DNA sequencing, are by their nature reliant on sequence similarity, and therefore limited to detect genes identical—or closely homologous—to known resistance genes [15]. However, numerous explorative studies of the resistance traits present in natural bacterial communities have revealed a vast range of resistance genes not (yet) found in human pathogens and which are thus unlikely to be annotated as resistance genes in sequence databases [16–20]. Among the human health risks associated with environmental antibiotic resistance [21], the arguably most severe one is the recruitment of novel resistance factors that are very rare or not yet present in human pathogens, because such genes could introduce new phenotypes to clinically relevant bacteria [22]. Since most of these resistance factors are unknown, this risk is impossible to quantify directly. Still, it may be possible to indirectly achieve a relative risk ranking of environments, based on other information regarding resistance [23]. In this context, it would be beneficial if we could use knowledge from large-scale quantification of antibiotic resistance genes to infer properties of this yet undescribed fraction of the environmental resistome. It is often assumed that the abundance and diversity of known resistance genes are representative also of the non-characterized fraction of the resistome in a given environment [15]. The elephant in the room, though, is whether this assumption is valid. In this study, the hypothesis that the diversity and abundance of known antibiotic resistance genes can predict the diversity and/or abundance of their undescribed counterparts is tested, by quantifying resistance genes across 864 samples from various environments [13], and 245 samples from the Tara Oceans project [24].

## Results

### Subsets of antibiotic resistance genes describe total gene diversity

**Fig. 1:**
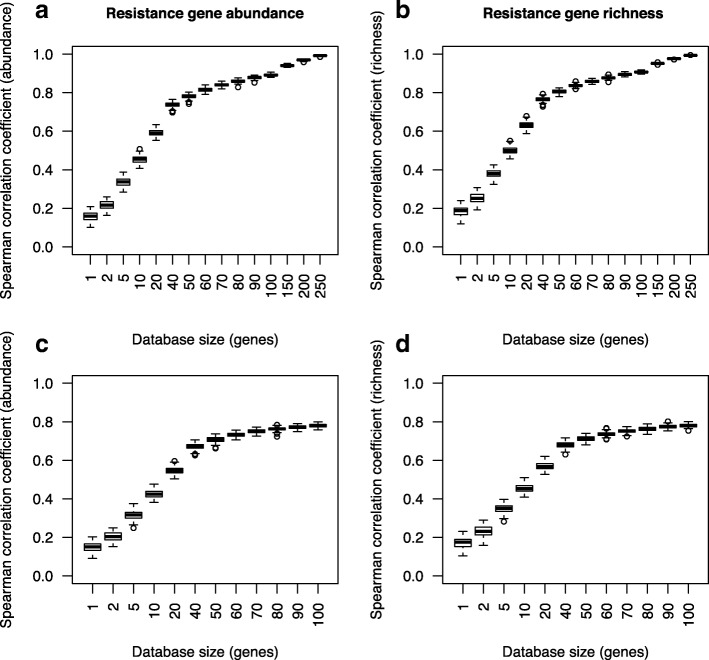
Predictive ranking power of randomly selected subsets of resistance genes on the full database. **a** Resistance gene abundance predictions when subsets were included in the full database. **b** Resistance gene richness predictions when subsets were included in the full database. **c** Abundance predictions when subsets were removed from the full database. **d** Richness predictions when subsets were removed from the full database

To test the ability of smaller sets of resistance genes to accurately rank environmental samples in terms of total resistance gene abundance and diversity, subsets of genes were randomly sampled from a database of known mobile antibiotic resistance genes, and their Spearman correlations to the entire set of genes in the database were calculated. In real-world scenarios, the genes in the resistance gene database would contribute to total resistance abundance, as well as genes not present in the database. To simulate this scenario, the subsamples of genes were included in the total dataset, and on average, a subset containing only 60 randomly selected genes (18% of the total database) could rank the resistance gene abundance in environmental samples in a way that correlated well (Spearman correlation > 0.8) with the ranking achieved from the full database (Fig. 1a). In terms of sample richness, only 50 genes (15% of the database) were required to achieve a correlation better than 0.8 to the ranking using the full database (Fig. 1b). To achieve a richness correlation above 0.9, 100 genes were required, and this value will be referred to as *P*_0.9_ = 100 in the following text, to allow for comparisons of prediction performance. For abundance, the *P*_0.9_ was 110 across all environments.

To some degree, this correlation is due to the fact that the most predictive genes are more likely to be present in the subsample as the size of the subsample grows. To compensate for this effect, a smaller subset was set aside from the database and used to predict the abundance and diversity of the remaining genes across all environments. In this case, both diversity and abundance estimates plateaued at a correlation coefficient around 0.75, which was reached at a subsample size of 50–60 resistance genes (Fig. 1c, d).

### Ranking of resistance gene diversity is valid across most environmental types

**Fig. 2:**
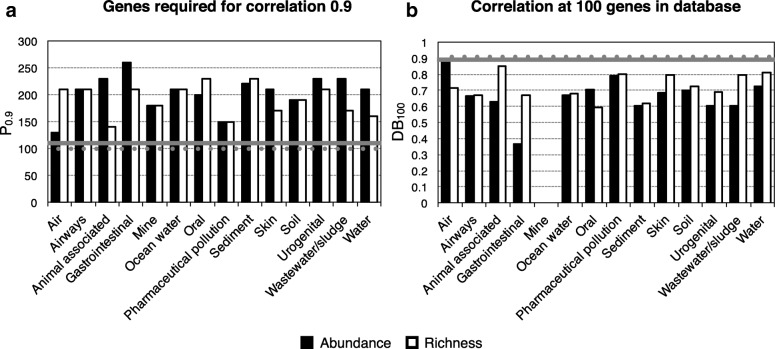
Predictive ranking power across environments for both resistance gene abundance and diversity. **a** Average number of genes required to obtain a Spearman correlation of 0.9 between the subset and the full resistance gene database. **b** Average Spearman correlation coefficient obtained between a subset of 100 randomly selected genes and the full resistance gene database. The horizontal lines represent *P*_0.9_ and DB_100_ across all environments for abundance (solid lines) and richness (dotted lines)

Different environments rank very differently in terms of resistance gene diversity and abundance [13], and to investigate if there was such an environmental bias in the data, the same procedure was repeated within each environmental type (Fig. 2). Although no environment type obtained a *P*_0.9_ below 110 (neither for abundance nor for richness; Fig. 2a), there were several environments that still retained high correlations using a subsample of 100 resistance genes (Fig. 2). The only instance with a Spearman correlation coefficient below 0.5 was the gastrointestinal sample abundance ranking (with the exception of the mine samples which generated too few resistance gene detections to allow for a correlation at 100 genes). At the same time, the richness ranking of the gastrointestinal samples had a correlation above 0.75 using subsamples of 100 genes.

### Some diversity measures describe the total diversity of resistance genes remarkably bad

It is unclear which diversity measures that would be most appropriate for estimating total resistance gene diversity from metagenomic sequencing [15]. However, testing the prediction power of six different measures (total abundance, gene richness, Shannon diversity, Simpson diversity, the Chao1 estimator, and the ACE estimator), it became clear that certain diversity measures are unsuitable for the task (Additional file 1: Figure S1). Using richness as a baseline (Additional file 1: Figure S1b), the Shannon index performed reasonably well in ranking the environmental samples, but was worse than the simpler richness measure (Additional file 1: Figure S1c). In contrast, the Simpson index showed low ranking performance even at a 100-gene subsample (Additional file 1: Figure S1d). Finally, the Chao1 estimator performed very similarly to richness (Additional file 1: Figure S1e), while the ACE estimator showed large fluctuations at low subsample sizes, but plateaued at about the same number of included genes as the richness did (Additional file 1: Figure S1f).

When the database was divided into a smaller subsample prediction set and a larger “true result” set, the Chao1 estimator still predicted the diversity about as well as the gene richness did, while the Shannon index showed slightly lower performance (Additional file 1: Figure S2). The Simpson index, however, performed remarkably poorly in this setting, not even reaching a correlation of 0.2 at a 100-gene subsample (Additional file 1: Figure S2d). Similarly, the ACE estimator showed very large fluctuations in its ranking performance, particularly at low subsample sizes (Additional file 1: Figure S2f).

### Small sets of selected resistance genes describe the total diversity remarkably well

**Fig. 3:**
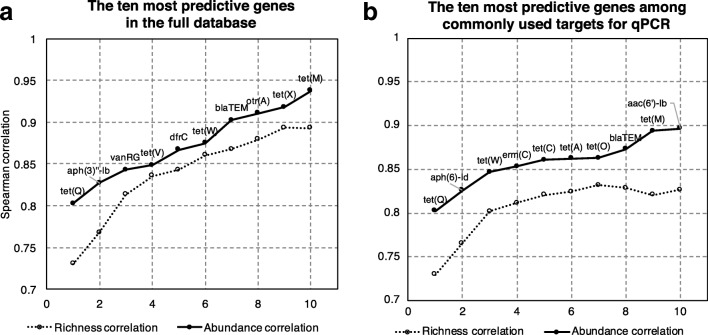
Most predictive resistance genes. Cumulative prediction power, expressed as the Spearman correlation for abundance and richness between the total database and the gene subset for the one to ten most predictive resistance genes in the full database (**a**), and a subset of resistance genes commonly suggested for qPCR monitoring (**b**), cumulatively combined

**Fig. 4:**
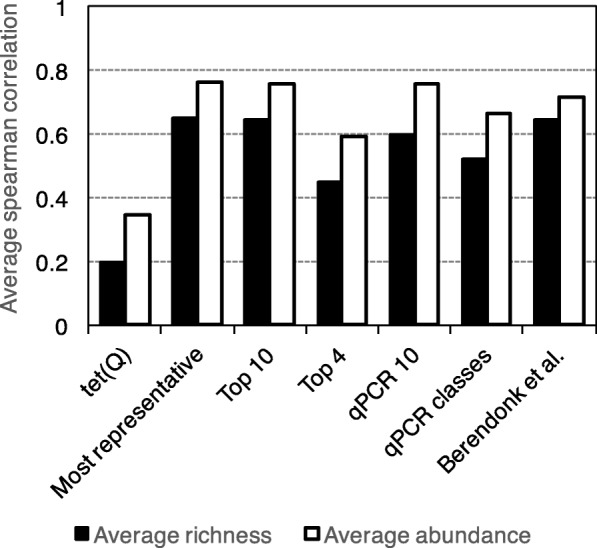
Average Spearman correlation between environments for different subsets of resistance genes. The *tet(Q)* gene represents the most predictive resistance gene overall, the “most representative” set are the genes that are found to be among the most predictive in at least three environments, and the “top 10” and “top 4” sets represent the ten and four most predictive resistance genes, respectively. The “qPCR 10” set consists of ten of the most commonly used genes in qPCR studies of environmental antibiotic resistance, and the “qPCR classes” set represents a set where one gene was selected from each antibiotic class among the most common qPCR targets. The “Berendonk et al.” set represents genes suggested for environmental monitoring by Berendonk et al. [25]

While it was clear from the above analysis that an incomplete set of resistance genes can predict the total abundance and diversity reasonably well, it was also clear from the underlying data that some genes contributed more to this prediction performance than others. Therefore, genes were systematically tested for combinations that yielded the highest possible correlation to the results of the full database, using as few genes as possible. Already by selecting a single resistance gene—*tet(Q)*—a correlation to the ranks obtained from total abundance of 0.80 was achieved, and for richness, the correlation using only *tet(Q)* was 0.73 (Fig. 3a). Further addition of genes raised the correlation to 0.94 for abundance and 0.89 for richness using only ten genes. Six out of these ten most predictive genes were tetracycline resistance genes, which speaks to the ubiquity of this resistance gene class across many environments. It should be noted that this high degree of precision may partially be due to the fact that certain genes are typical of certain environments, and that these ten genes may separate, e.g., human gut and environmental resistomes exceptionally well. To investigate the magnitude of this effect, the correlations between these genes and total diversity and abundance were investigated for all environments separately (Fig. 4). This showed that the top ten predictive genes on average had Spearman correlations to the entire resistance gene database of 0.65 for richness and 0.76 for total abundance. This is comparable to the predictive power of the 12 most representative (selected on the criterion of being one of the ten most predictive resistance genes in at least three environments), and substantially better than using the top four most predictive genes or only the best gene—*tet(Q)*—alone. However, for any given gene set, the variations between environments were fairly large, but with the same general tendency to achieve better predictions the more genes that were included in the predictive subset (Additional file 1: Figure S3). That said, in 13 of 14 environments, the ten most predictive genes were significantly predictive of richness, and they were predictive of total abundance in 12 (Additional file 2: Table S1). Finally, the performance of a set of commonly selected resistance genes for qPCR-based studies of environmental resistance was investigated (Table 1; Additional file 3: Table S2). In general, the top ten predictive genes identified in this study performed slightly better than the ten most commonly used genes for qPCR did (Figs. 3 and 4), although the latter, when used together, were also significantly predictive for richness in 13 and abundance in 12 environments (Additional file 2: Table S1). The top ten genes identified in this study also performed slightly better than the genes suggested for surveillance by Berendonk et al. [25]. Regardless, a common feature among the most predictive genes was that they included *tet(Q)*, an aminoglycoside resistance gene (*aph(3)″-Ib* or *aph(6)-Id*) and the *bla*_TEM_ beta-lactamase (Fig. 3), which could be considered a minimal subset of genes to have a reasonable chance of describing resistance gene diversity.

**Table 1:** Predictive performance measured as Spearman correlation for resistance genes commonly used for studies employing qPCR on the richness and abundance of all Resqu and FARME genes

		Abundance prediction		Richness prediction		Average correlation
Gene name		Resqu	FARME	Resqu	FARME	
*tet(Q)*		0.80	0.65	0.73	0.04	0.56
*tet(O)*		0.75	0.63	0.70	0.08	0.54
*tet(W)*		0.78	0.62	0.72	0.04	0.54
*erm(F)*	*	0.57	0.44	0.60	0.08	0.42
*erm(B)*	*	0.52	0.38	0.52	0.02	0.36
*tet(M)*	*	0.56	0.23	0.53	− 0.11	0.30
*sul2*	*	0.30	0.24	0.39	0.22	0.29
*aph(6)-Id*	**	0.27	0.17	0.36	0.16	0.24
*sul1*	*	0.21	0.16	0.30	0.21	0.22
*tet(G)*		0.20	0.14	0.32	0.21	0.22
*tet(A)*		0.21	0.16	0.29	0.18	0.21
*aac(6′)-Ib*	**	0.14	0.13	0.23	0.26	0.19
*tet(C)*		0.18	0.10	0.25	0.20	0.18
*tet(S)*		0.15	0.11	0.23	0.15	0.16
*sul3*		0.13	0.11	0.16	0.08	0.12
*qnrS*	*	0.12	0.10	0.13	0.07	0.10
*tet(B)*		0.19	0.11	0.19	− 0.06	0.10
*bla*_TEM_	*	0.10	0.01	0.11	0.14	0.09
*tet(E)*		0.08	0.06	0.11	0.10	0.09
*erm(C)*		0.11	−0.05	0.19	− 0.05	0.05
*bla*_CTX-M_	*	0.00	0.00	0.00	0.00	0.00
*bla*_KPC_	**	0.00	0.00	0.00	0.00	0.00
*bla*_NDM_	**	0.00	0.00	0.00	0.00	0.00
*bla*_VIM_	**	0.00	0.00	0.00	0.00	0.00

Genes with an asterisk were suggested by Berendonk et al. (2015) [25]. Genes with two asterisks were suggested by Berendonk et al. but are not commonly employed in qPCR studies

### Known mobile resistance genes can predict the diversity of recently discovered ones

**Fig. 5:**
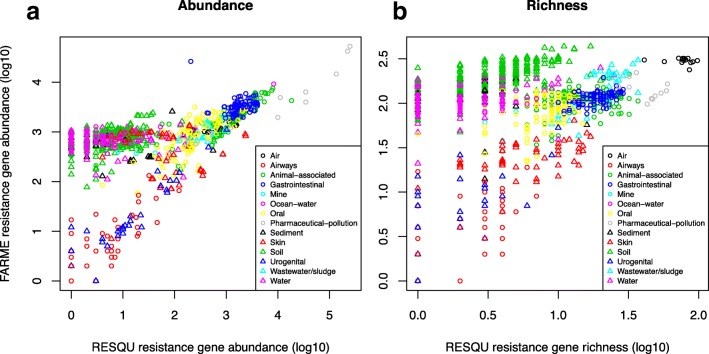
Predictive power of known resistance genes on novel ones. Relationships between the measurements made based on Resqu genes and FARME genes, in terms of **a** total resistance gene abundance and **b** resistance gene richness

Investigating the relationships between abundance and diversity obtained from subsets of resistance genes and the entire database provides for controlled conditions in which the true expected answer to the predictions made is known. However, this type of evaluation does not fully reflect the actual complexity of environmental antibiotic resistance. It could be assumed, for example, that mobile resistance genes have originated on bacterial chromosomes and that the vast majority of resistance genes are not yet described [8]. To provide an external validation of the findings based on the Resqu database, which only contains resistance genes identified on mobile genetic elements, the same samples were also analyzed for resistance genes using the FARME database—a repository of genes from functional metagenomics inserts providing antibiotic resistance. These genes represent a set of true resistance genes with very different degree of similarity to the genes in the Resqu database and form an ideal testing set for the predictions made using the latter. Overall, predictions made from the entire Resqu database corresponded fairly well to the total resistance gene abundance and richness obtained from the FARME database (Fig. 5; Tables 1 and 2; Spearman correlation 0.31 for richness, 0.62 for abundance). Interestingly, those levels of correlations were reached already at a subset size of 40 to 50 Resqu genes (Additional file 1: Figure S4). Linear models based on Resqu were significantly predictive in most environments (12/14), but their performance was not equally good (Table 2). Particularly, the models were not significantly predictive for either richness or abundance in sediment and mine samples. The mine environmental type had the least numbers of samples (seven), which may explain the lack of significance, but 45 sediment samples were included in this study, making small sample size a less likely explanation in this case.

**Table 2:** Adjusted *p* values for predictiveness of Resqu genes on the richness and abundance of FARME genes

	All samples	Animal-associated	Sediment	Wastewater/sludge	Soil	Water	Air	Mine
Richness	5.3E−24	0.023	0.43	7.9E−06	3.3E−15	0.018	0.58	0.96
Abundance	2.9E−71	5E−33	0.48	1.0E−05	1.2E−09	0.96	4.4E−05	0.105
	***	*		**	***	*		
	***	***		**	**		**	
		Gastrointestinal	Oral	Airways	Urogenital	Skin	Pharmaceutical pollution	Ocean water
Richness		0.00034	0.00032	0.00065	4.7E−11	0.00065	0.31	9.3E−07
Abundance		0.95	3.1E−19	1.1E−05	3.3E−12	0.58	1.3E−05	1.2E−09
		**	**	**	***	**		**
			***	**	***		**	**

Asterisks denote significance levels; * : 0.05 > p > 0.01; ** : 0.01 > p > 1.0E-10; *** : 1.0E-10 > p

Next, the most predictive genes from the subset analysis were investigated for their ability to predict the total abundance and diversity of FARME resistance genes across environments. These genes were significantly related to total FARME richness and abundance in eight environments (Additional file 4: Table S3), while the top four genes were only predictive in six. The most representative subset of genes was predictive of richness in nine environments and of abundance in seven. This was comparable to the most commonly used genes for qPCR used in combination. However, across all environments, the commonly used qPCR genes were not predictive of resistance gene richness, while they were for total abundance (Additional file 4: Table S3). It should be noted that using the *tet(Q)* gene alone was not significantly predictive of total FARME richness (linear model *p* = 0.276), but was related to resistance gene abundance (*p* = 1.65 × 10^−36^). Similarly to what was shown in the subset analysis, the Shannon and Simpson diversity indices were poorer predictors of total diversity than the simpler richness measure (Additional file 1: Figures S4 and S5). At the same time, the Chao1 and ACE estimators based on the top ten genes from the Resqu data did decent predictions of total FARME richness, with particularly the Chao1 estimator showing a stronger relationship to FARME richness than Resqu richness did (Additional file 1: Figure S5).

### Diversity and abundance relationships

**Fig. 6:**
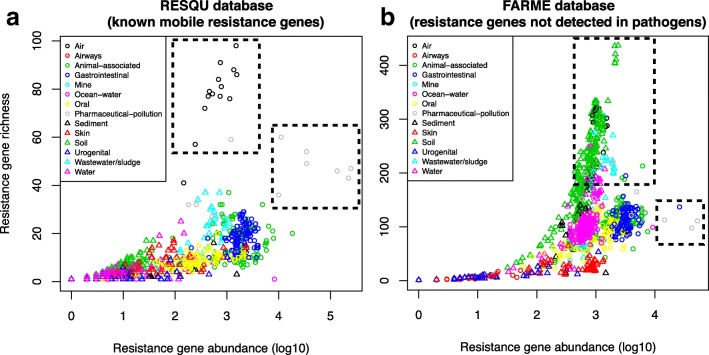
Relationships between abundance and diversity of resistance genes. **a** Abundance of known mobile resistance genes circulating in pathogens (Resqu database) compared to their richness across samples. **b** Abundance of resistance genes not yet detected in pathogens (FARME database) compared to their richness across samples. High-risk environments, i.e., high-richness or high-abundance settings, are indicated by dashed squares

Depending on how risks associated with antibiotic resistance gene findings are prioritized, different types of environments could be considered to be high-priority environments for mitigation [21, 22, 25, 26]. One important consideration in risk prioritization efforts is whether high abundance or high diversity of resistance genes in an environment poses the highest risk to human health [8]. Previous research using a more limited set of samples has suggested that environments with high abundance but low diversity of resistance genes are rarely encountered, while high diversity of resistance genes can be found without them being particularly abundant [27]. To confirm whether this holds true on a larger set of samples and environmental types, the abundance-diversity relationship was investigated for both the Resqu and FARME databases (Fig. 6). Interestingly, this analysis highlighted that for known mobile resistance genes already circulating in pathogens, there does not seem to be a requirement for a sample to have a high diversity of resistance genes to also show high abundances. For resistance genes identified from functional metagenomics studies, i.e., mostly not detected in human pathogens, the picture was somewhat different, with a clearer relation between high diversity and higher abundance of resistance genes (Fig. 6b). Here, particularly the soil samples stood out as having richness as a strong driver of abundance, while human-associated samples (gut, skin, oral, urogenital) showed no such relationship. Notably, some of the samples from environments polluted by pharmaceutical production waste had markedly higher abundance of resistance genes not yet been found in human pathogens, indicating the potential these environments have to mobilize such genes and make them available to pathogens in the future [8].

## Discussion

Calls for monitoring antibiotic resistance in the environment have been frequently made in the recent past [25, 28–34]. Such monitoring schemes are limited to targeting genes that are already known, and generally, a subset of genes known to cause clinical problems are investigated [25]. However, while the detection of genes already circulating among human pathogens is indicative of environmental dissemination of resistant bacteria, this may not always be the scenario that would be most relevant to monitor. In comparison, the emergence of novel resistance genes in pathogens may be the most severe long-term consequence of environmental selection for antibiotic resistance [8, 22]. This raises the question if resistance genes found in clinically relevant bacteria can serve as a proxy for the unknown resistance determinants in environmental bacterial communities and could therefore be used to rank environments in terms of risks to human health, regardless of perspective. The results of this study imply that recruitment of resistance genes from the environment is essentially stochastic. Therefore, given a sufficiently large subset of known resistance genes, the total abundance and diversity of all resistance determinants can be predicted and environments can be ranked accurately, at least in most cases. Importantly, the required size of the subset is largely determined by how the set of genes to be investigated is selected. If selected at random, at least 40–50 resistance genes were needed to get a good predictive power for ranking, while if the genes were selected systematically, a subset of only three or four genes could predict total abundance and diversity fairly well. This means that even if the currently used resistance gene databases, such as CARD [35] and ResFinder [36], lack a vast majority of the resistance genes present in nature [15], the findings obtained using them can still guide risk ranking based on metagenomic sequencing data.

In terms of implications for risk management, this suggests that environments with a high diversity and/or a high total abundance of resistance genes are good first candidates for mitigation efforts. This would highlight the importance of environments subjected to pollution from pharmaceutical production, as those were measured to contain the largest numbers of resistance genes, both known mobile and “novel” ones from the FARME database. The latter ones are derived from functional metagenomics studies identifying resistance genes not yet encountered in pathogens and may thus reflect a future potential to be recruited into human pathogens. Soil thereby seems to be the most important source of future resistance genes of all environments investigated. However, this could partially be due to a database bias in FARME, as soil has been one of the most common types of substrates used for functional metagenomics studies. Hence, genes from typical soil bacteria may be over-represented in the database [37, 38]. In any case, the results of this study again emphasize that the main risks in terms of recruitment of resistance factors from environmental reservoirs would be milieus exposed to relevant levels of antibiotics [39], including those exposed to waste from pharmaceutical production [9, 40], animal agriculture [41, 42], and untreated sewage [43, 44]. That said, monitoring of critically important resistance genes, such as the NDM and VIM beta-lactamases and *mcr*-1, can still be highly valuable for informing risk management related to dissemination of resistant bacteria through the environment, despite that these genes carry limited information on total resistance gene diversity in environmental settings.

A simpler and cheaper alternative to metagenomic sequencing often employed for monitoring the presence of antibiotic resistance genes in the environment is qPCR. The findings of this study have several important implications for qPCR-based monitoring efforts. First, the selection of genes investigated is critical for how well findings can be extrapolated from the tested gene set to the overall total abundance and diversity of resistance genes in a given environment. If genes are picked at random (or without prior knowledge), at least 30–40 genes would be required to rank environments with a reasonable accuracy, while a careful selection of genes brings this number down to about ten (Fig. 3). Below ten, the predictive power becomes poorer, meaning that regardless of which resistance genes that are chosen, studies employing single digit numbers of genes to infer resistance gene diversity or abundance are rather likely to be wrong. However, when a larger set of commonly used targets for qPCR are used together, their predictive performance is almost as good as when genes are selected by observed predictive performance in this study (Fig. 3). The targets proposed by Berendonk et al. [25] are almost equally predictive, given that at least ten of them are used together, even though their individual performances are quite poor (Table 1). It should also be noted that the higher the abundance and diversity of the investigated set is in an environment, the more accurate is its prediction of the total abundance and diversity.

In contrast, qPCR arrays utilizing hundreds of target genes to estimate abundance and diversity [12, 41, 45] are likely to be perfectly fitted for monitoring tasks. Already for a subset of 40 genes, the prediction performance for ranking environments was good, and at 100 genes abundance and diversity were recaptured almost as well as when the entire database was used. These arrays are less costly than performing a full shotgun metagenomics experiment. However, using metagenomics has the upside of providing a plethora of additional data [15], offering the potential to analyze the data for taxonomy [46], genes important for dealing with other types of stressors such as biocides and metals [47, 48], horizontal gene transfer capacity [49], or metabolic pathways [50]. Moreover, metagenomic sequencing enables reanalysis of sequence data for new resistance genes discovered after the samples were initially analyzed [51, 52], enabling retrospective analysis of monitoring data. An additional benefit of utilizing metagenomic sequencing over qPCR arrays is the possibility to computationally predict novel resistance genes from sequence data [53, 54], although this specific practice is quite unlikely to be employed as part of monitoring schemes.

While smaller subsets of resistance genes are overall predictive of total resistance gene abundance and diversity, their prediction power is not equal across environments. For example, while most gene subsets performed well in animal-associated environments, the variation in prediction performance in wastewater/sludge samples was substantial. Interestingly, the gene sets often used or proposed for qPCR were better predictors of total abundance in wastewater samples than the gene sets identified to be most predictive in this study. This was in contrast to most other environments and likely reflects a bias in where resistance gene abundances have been studied the most [43]. Moreover, it is notable that while the abundance and diversity of resistance genes in environments exposed to pollution with pharmaceutical waste were fairly predictable, the abundance of resistance genes in the human gut was rather difficult to predict—much harder than predicting similar metrics in animal-associated samples. Unfortunately, it seems that the type of environment studied matters for the power to extrapolate to the total abundance and diversity of resistance genes and furthermore that it matters in a fairly unpredictable way. This highlights the continued need for further characterization of novel resistance factors and investigations of a wide range of resistance genes across diverse environments.

The most appropriate measure for approximating resistance gene diversity has been debated, and there is currently no clear consensus on which method that is preferable [15]. This study shows very clearly that there are some methods that should be ruled out, because they render inaccurate predictions and perform poorly in terms of ranking environments. For example, the Simpson diversity index consistently showed poor performance, particularly when Resqu data was used to estimate the diversity of FARME genes. The Shannon index performed relatively better, but there is still no reason to select the Shannon index over normalized (rarefied) richness of resistance genes. As shown before, the ACE estimator fluctuates substantially compared to the other diversity measures [15], while the Chao1 estimator more consistently showed performance very similar to richness. In addition, Chao1 was slightly better at predicting the total diversity from a small subset of genes. The bottom line is that either richness or Chao1 could be used with virtually the same ranking results, while the Shannon, Simpson, and ACE measures should clearly be avoided for estimating resistance gene diversity.

## Conclusions

This study shows that the diversity and abundance of known antibiotic resistance genes can generally predict the diversity and abundance of undescribed resistance genes, although to what degree is dependent on the type of environment investigated and likely also many other parameters that were not measured in this study. This implies that the recruitment of novel antibiotic resistance genes from the environment to human pathogens is essentially random. Therefore, when ranking risks associated with antibiotic resistance in environmental settings, the knowledge gained from large-scale quantification of known resistance genes can be utilized as a (sometimes coarse-grained) proxy for the unknown resistance factors. Thus, milieus previously pointed out as high-risk environments for resistance development and dissemination based on broad screens for resistance genes remain the most likely to be important, including aquaculture, animal husbandry, discharges from antibiotic manufacturing, and untreated sewage [2, 8, 28, 55–57]. Further attention should probably be paid to antibiotic contaminated soils, as soils seem to be a vast source of resistance genes not yet encountered in human pathogens, as has also been suggested previously [4, 16, 58, 59]. Soil, however, is a globally present, very diverse habitat with microbial composition varying with biochemical properties and geographical gradients [60, 61], setting practical barriers for mitigation efforts aside from avoiding contamination of soils with antibiotics. The results of this study can be used to guide monitoring efforts for environmental antibiotic resistance, to design risk ranking strategies, and to choose appropriate measures and methods for describing resistance gene abundance and diversity in the environment.

## Methods

### Dataset and database selection

To obtain a large number of samples that had both been sequenced using the same methodology and had a coherent environmental classification, this study utilized the datasets selected by Pal et al. [13], with the addition of 245 samples from the Tara Oceans project [24]. These 1109 samples were all sequenced using Illumina technology and had a sequencing depth covering at least 10 million reads per metagenome (Additional file 5: Table S4). As a database representing well-known, mobile antibiotic resistance genes, Resqu was selected (version 1.1; http://www.1928diagnostics.com/resdb/ [9]). Resqu contains 3018 non-redundant protein sequences divided into 325 resistance gene types, all reported to have been horizontally transferred between at least two different bacterial species and conferring a verified resistance phenotype. This database was contrasted against FARME [38], a repository of genes found on inserts confirmed to provide antibiotic resistance based on functional metagenomics [62]. Some of these genes represent true resistance genes (with different degree of similarity to the genes in the Resqu database), forming an ideal testing set for the predictions made based on subsets of genes. However, the FARME database contains every gene found in the inserts from functional metagenomics, and hence, many of the sequences in FARME are not actual resistance genes. To circumvent this problem, the database was filtered according to the following. First, the protein sequences (26,253 in total) and the HMM analysis table were downloaded from the FARME website (http://staff.washington.edu/jwallace/farme/download.html) on 2017-02-16. Then, only the proteins with an annotated antibiotic resistance function in the HMM analysis table (column “Antibiotic Resistance”) were extracted from the protein FASTA file (4432 sequences). These were clustered into 90% identity clusters using Usearch [63] to reduce redundancy, resulting in 2612 non-redundant resistance genes used for the rest of the study (Additional file 6).

### Resistance gene quantification

To make all metagenomes comparable, every library was randomly subsampled to 10 million reads using a custom Perl script. The datasets were analyzed similar to in Pal et al. [13]. Each subsampled library was searched against the Resqu and FARME databases using Usearch (v8.0.1445) with a sequence identity threshold of 90% (options “-usearch_global -id 0.9 -maxaccepts 1 -threads 16”). Hits were organized into resistance gene types according to gene mapping files, and abundance matrices of raw counts were constructed using metaxa2_dc [64].

### Statistical analysis

All statistical analyses were carried out in R version 3.3.2 [65] with the additional packages vegan version 2.4-1 [66] and gplots version 3.0.1 [67]. For each sample, “true” target values were calculated for the sum of all resistance gene counts (total abundance), the richness of resistance genes (the number of different gene types found), the Shannon diversity index [68], the Simpson diversity index [69], the Chao1 estimator [70], and the ACE estimator [71]. Next, the database was subsampled to contain only a subset of the resistance genes, and the same values were recomputed for all samples. The values for the subsampled database were compared to the corresponding values obtained using the full database using the Spearman rank correlation. The subsampling procedure was repeated 100 times, both for the full dataset and for each environmental type separately. Two measures were defined to describe prediction power: the *P*_0.9_, which was defined as the smallest number of genes needed to achieve a correlation above 0.9, and the DB_100_, which was defined as the correlation coefficient obtained using 100 resistance genes in the database.

To identify the most predictive subset of resistance genes in the database, the gene with the best correlation (average of richness and abundance correlations) to the total database was selected and rerun in combination with every gene in the database. The pair with the best correlation was selected and again rerun in combination with every gene in the database to find the best combination of three. This procedure was repeated until the ten most predictive resistance genes had been identified, both in all samples together and in each environmental type separately. This was compared to the correlations obtained by pre-selected subsets of genes, e.g., those most commonly used in qPCR studies of resistance in the environment (Additional file 3: Table S2). The predictiveness of each subset on the full database was assessed using a linear model with the subset-derived values as explanatory variables. A subset was considered to be significantly predictive when its Benjamini-Hochberg-adjusted [72] *p* value for zero slope was below 0.05. Abundance values were log-transformed before inclusion in the linear models.

Finally, the entire analysis was repeated using the results obtained from the FARME database as the “true” expected result. The same measures (for FARME data) were calculated for each sample, and Spearman rank correlations to the results obtained from Resqu (above) were investigated. The predictiveness of the full database as well as the subsets of resistance genes were assessed using linear models, as described above. The R scripts used for the analysis are available in Additional file 7.

## Additional files

Additional file 1: **Figure S1.** Predictive ranking power of randomly selected subsets of resistance genes on the full database, when subsets were included among the genes in the full database. A) Resistance gene abundance. B) Resistance gene richness. C) Shannon diversity index. D) Simpson diversity index. E) Chao1 estimator. F) ACE estimator. **Figure S2.** Predictive ranking power of randomly selected subsets of resistance genes on the full database, when subsets were excluded from the full database. A) Resistance gene abundance. B) Resistance gene richness. C) Shannon diversity index. D) Simpson diversity index. E) Chao1 estimator. F) ACE estimator. **Figure S3.** Average Spearman correlation across environments for different subsets of resistance genes. **Figure S4.** Predictive ranking power of randomly selected subsets of resistance genes in Resqu on the full FARME database. A) Resistance gene abundance. B) Resistance gene richness. C) Shannon diversity index. D) Simpson diversity index. E) Chao1 estimator. F) ACE estimator. **Figure S5.** Relationships between the measurements made based on the ten most predictive resistance genes in Resqu and the total set of FARME genes, in terms of A) total resistance gene abundance, B) resistance gene richness, C) the Shannon diversity index, D) the Simpson diversity index, E) the Chao1 estimator, and F) the ACE estimator. (PDF 693 kb)

Additional file 2: **Table S1.** Significant predictive power of different subsets of resistance genes on known mobile antibiotic resistance genes in Resqu. (XLSX 11 kb)

Additional file 3: **Table S2.** Commonly used target genes for qPCR-based studies of resistance genes in the environments collected from the literature. (XLSX 24 kb)

Additional file 4: **Table S3.** Significant predictive power of different subsets of resistance genes on antibiotic resistance genes not yet detected in pathogens (FARME database). (XLSX 11 kb)

Additional file 5: **Table S4.** Complete list of all datasets used for this study. (XLSX 29 kb)

Additional file 6: The non-redundant resistance genes in the filtered version of the FARME database used for this study. (FASTA 670 kb)

Additional file 7: The R scripts used for the analysis in this study. (ZIP 18 kb)

## Abbreviations

DB_100_: The smallest number of genes needed to achieve a Spearman correlation above 0.9
HMM: Hidden Markov model
*P*_0.9_: The Spearman correlation coefficient obtained using a database of 100 resistance genes
qPCR: Quantitative real-time polymerase chain reaction
WHO: World Health Organization
