# Supplementary material for: The diversity of uncharacterized antibiotic resistance genes can be predicted from known gene variants—but not always
Source: Microbiome. 2018 Jul 7;6:125. doi: 10.1186/s40168-018-0508-2 (PMC6035801; doi:10.1186/s40168-018-0508-2)
Supplement: Supplementary file 1 — Figure S1. Predictive ranking power of randomly selected subsets of resistance genes on the full database, when subsets were included among the genes in the full database. A) Resistance gene abundance. B) Resistance gene richness. C) Shannon diversity index. D) Simpson diversity index. E) Chao1 estimator. F) ACE estimator. Figure S2. Predictive ranking power of randomly selected subsets of resistance genes on the full database, when subsets were excluded from the full database. A) Resistance gene abundance. B) Resistance gene richness. C) Shannon diversity index. D) Simpson diversity index. E) Chao1 estimator. F) ACE estimator. Figure S3. Average Spearman correlation across environments for different subsets of resistance genes. Figure S4. Predictive ranking power of randomly selected subsets of resistance genes in Resqu on the full FARME database. A) Resistance gene abundance. B) Resistance gene richness. C) Shannon diversity index. D) Simpson diversity index. E) Chao1 estimator. F) ACE estimator. Figure S5. Relationships between the measurements made based on the ten most predictive resistance genes in Resqu and the total set of FARME genes, in terms of A) total resistance gene abundance, B) resistance gene richness, C) the Shannon diversity index, D) the Simpson diversity index, E) the Chao1 estimator, and F) the ACE estimator. (PDF 693 kb) [file 40168_2018_508_MOESM1_ESM.pdf]

The diversity of uncharacterized antibiotic resistance genes can be predicted from known gene variants – but not always

Johan Bengtsson-Palme[1,2,3,*]

Supplementary Material

Affiliations
[1] Department of Infectious Diseases, Institute of Biomedicine, The Sahlgrenska Academy, University of Gothenburg, Guldhedsgatan 10, SE-413 46, Gothenburg, Sweden

[2] Centre for Antibiotic Resistance research (CARe) at University of Gothenburg, Gothenburg, Sweden

[3] Wisconsin Institute for Discovery, University of Wisconsin-Madison, 330 North Orchard Street, Madison WI 53715, USA

* Correspondance: Johan Bengtsson-Palme, Department of Infectious Diseases, Guldhedsgatan 10, SE-413 46, Gothenburg, Sweden. Phone: +46 31 342 46 26. Fax: +46 31 84 61 13. E-mail address: johan.bengtsson-palme@microbiology.se

Figure S1.

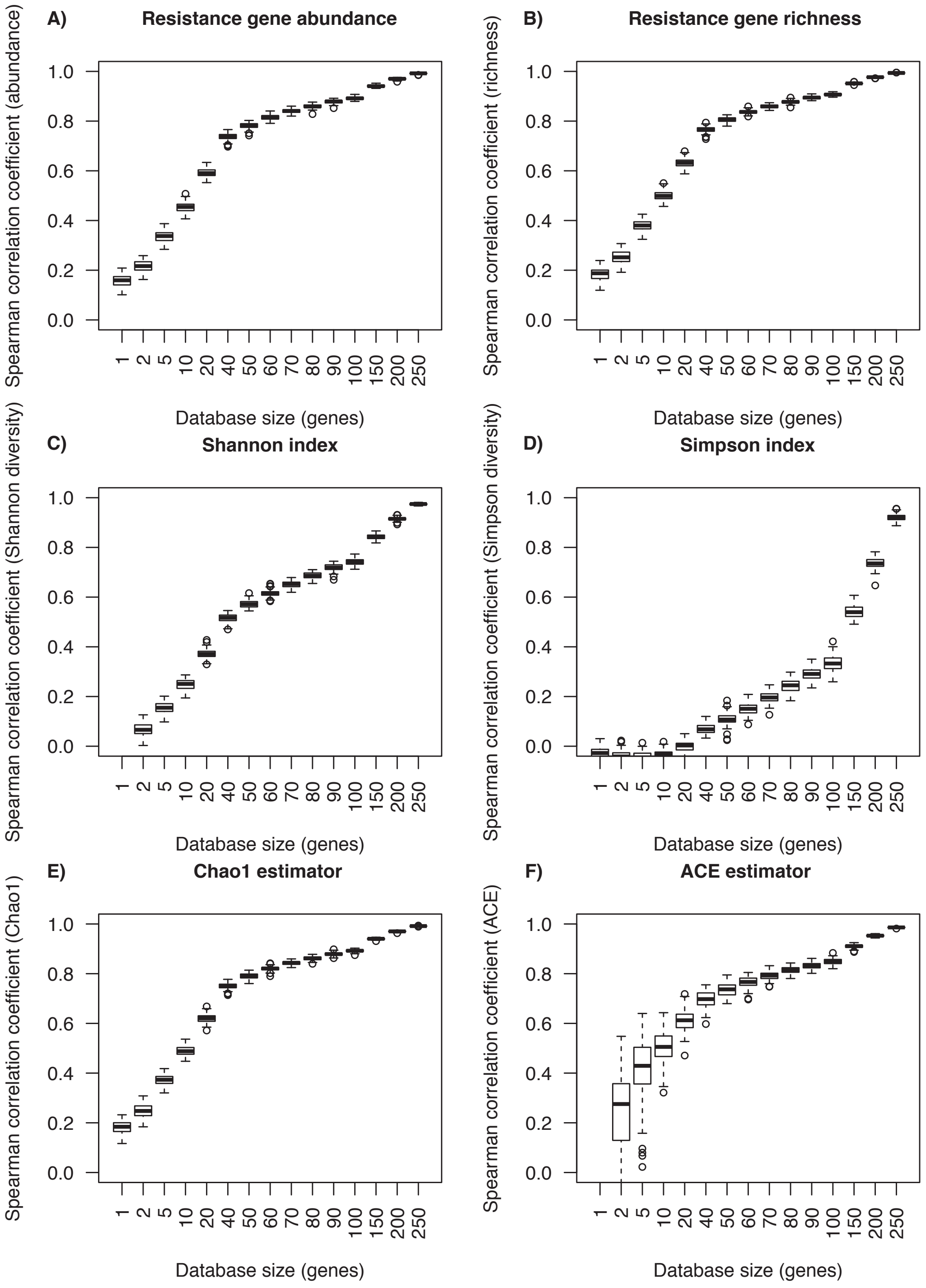

Fig. S1. Predictive ranking power of randomly selected subsets of resistance genes on the full database, when subsets were included among the genes in the full database. A) Resistance gene abundance. B) Resistance gene richness. C) Shannon diversity index. D) Simpson diversity index. E) Chao1 estimator. F) ACE estimator.

Figure S2.

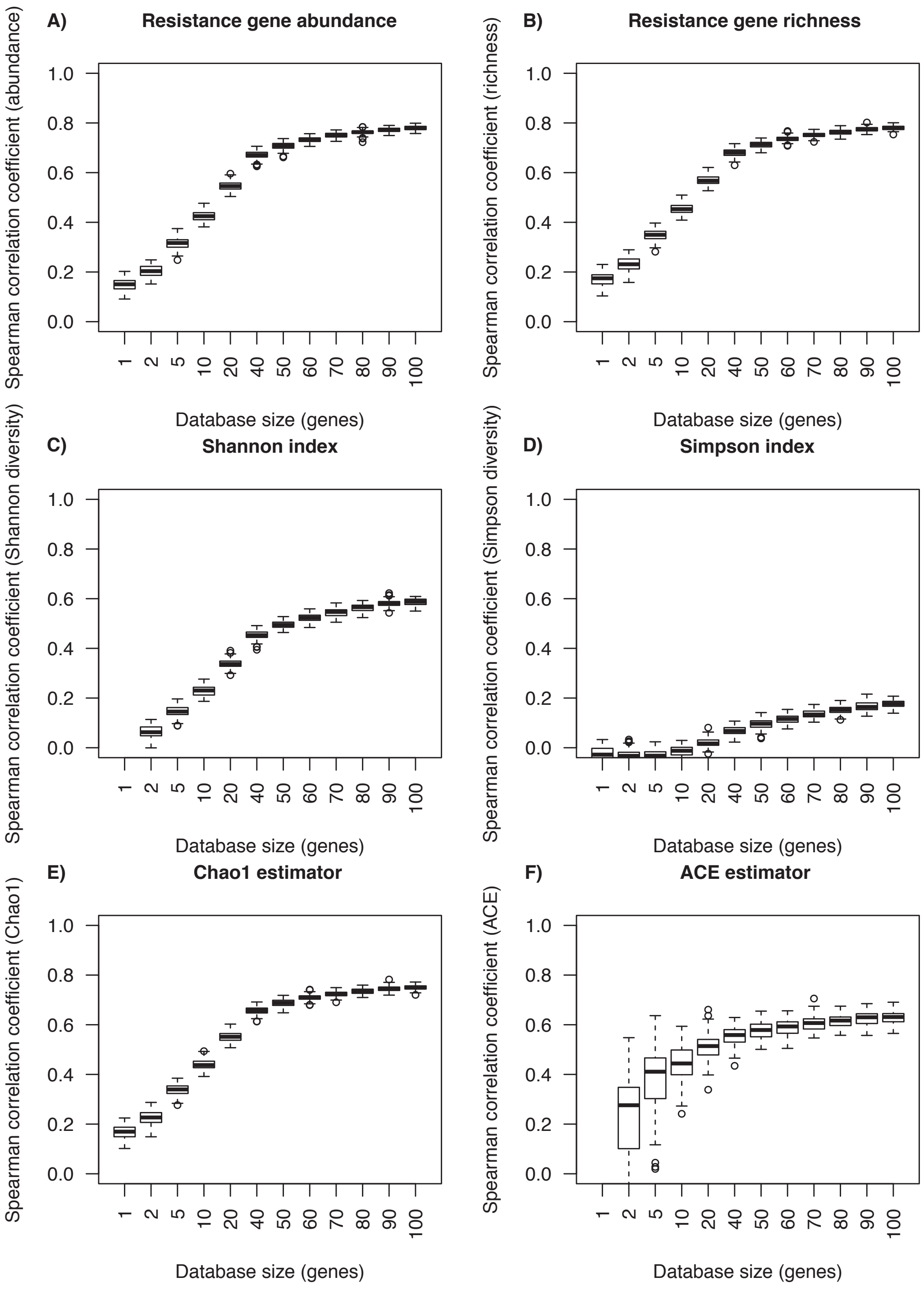

Fig. S2. Predictive ranking power of randomly selected subsets of resistance genes on the full database, when subsets were excluded from the full database. A) Resistance gene abundance. B) Resistance gene richness. C) Shannon diversity index. D) Simpson diversity index. E) Chao1 estimator. F) ACE estimator.

Figure S3.

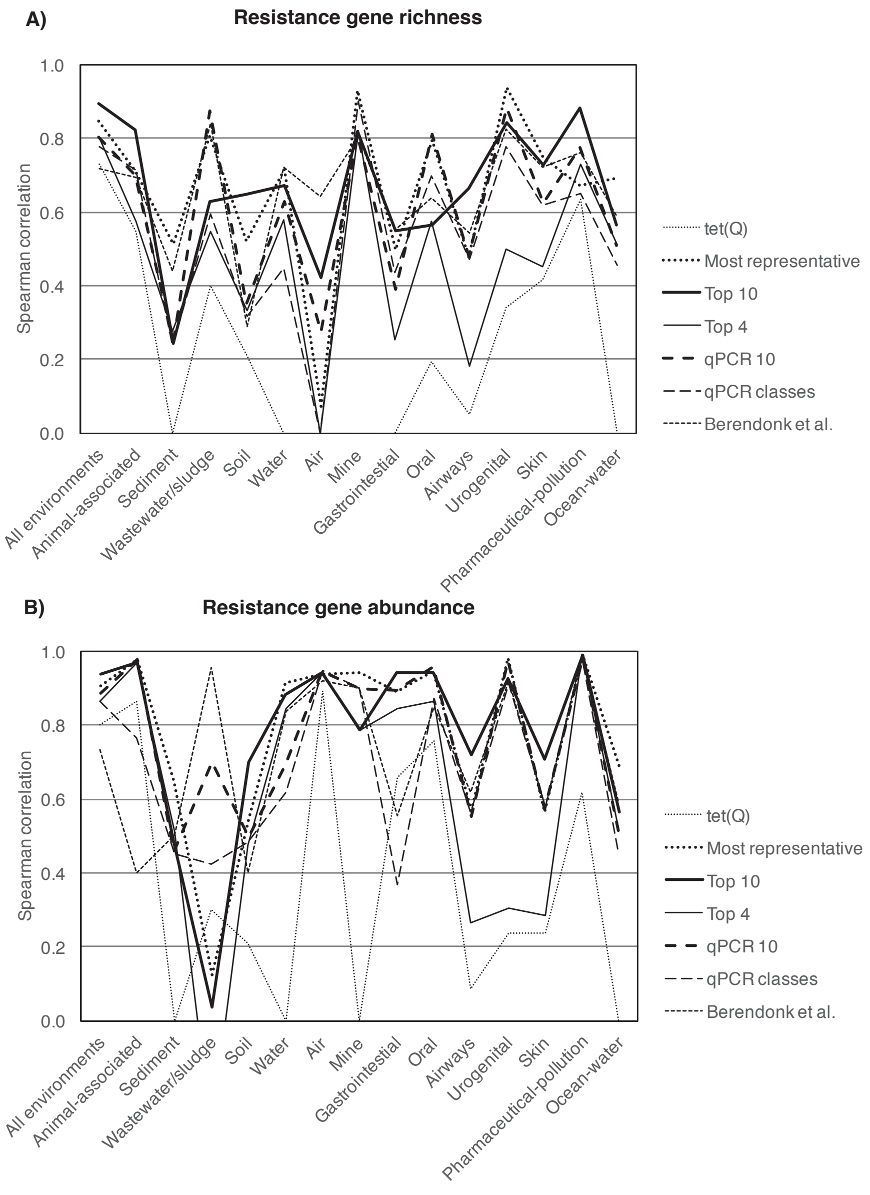

Fig. S3. Average Spearman correlation across environments for different subsets of resistance genes. The *tet(Q)* gene represents the most predictive resistance gene overall, the 'most representative' set are the genes that are found to be among the most predictive in at least three environments, and the 'top 10' and 'top 4' sets represent the ten and four most predictive resistance genes, respectively. The 'qPCR 10' set consists of ten of the most commonly used genes in qPCR studies of environmental antibiotic resistance, and the 'qPCR classes' set represents a set where one gene was selected from each antibiotic class among the most common qPCR targets. The 'Berendonk et al.' set represents genes suggested for environmental monitoring by Berendonk et al. [22]. A) Resistance gene richness. B) Resistance gene abundance.

Figure S4.

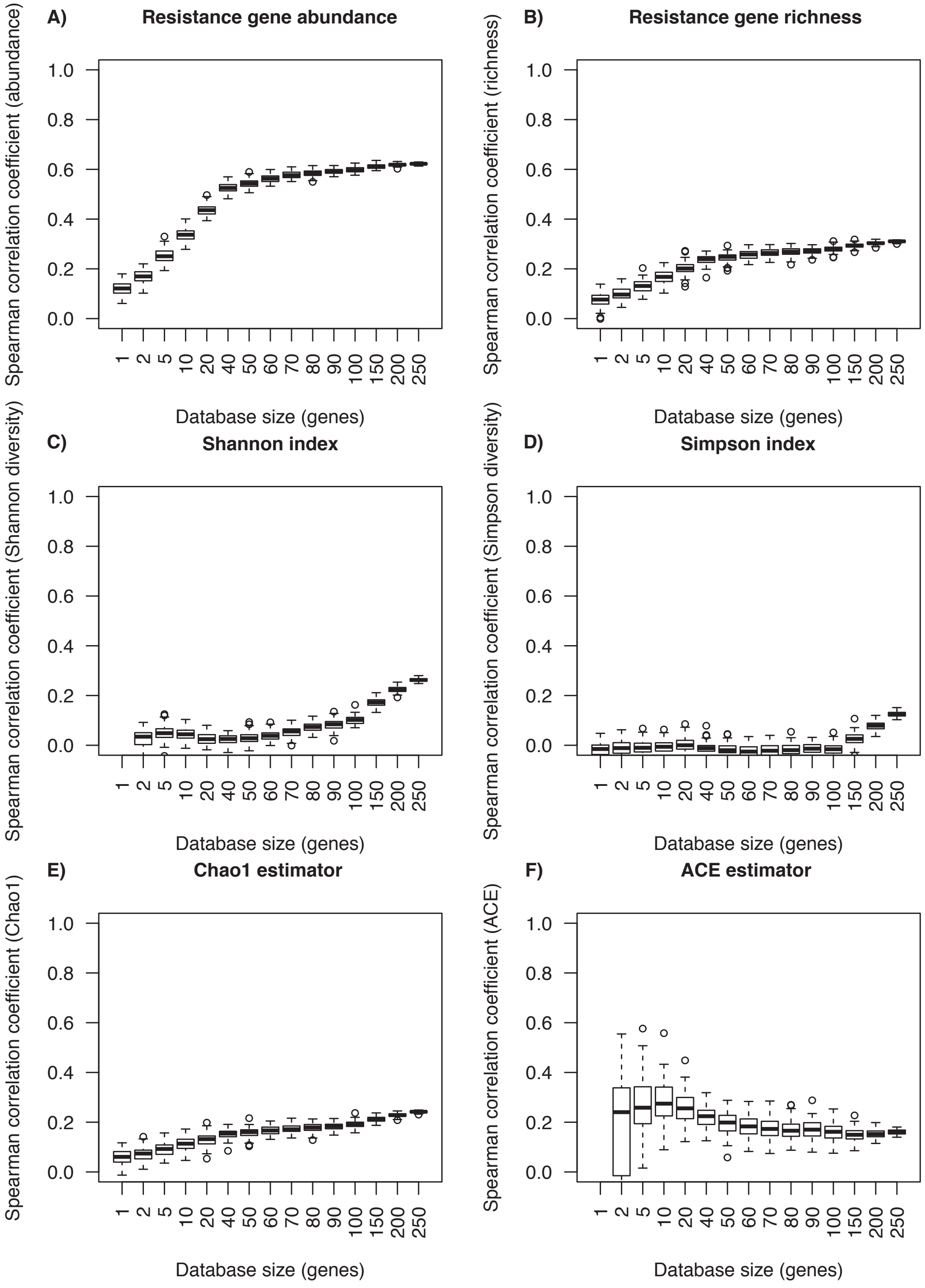

Fig. S4. Predictive ranking power of randomly selected subsets of resistance genes in Resqu on the full FARME database. A) Resistance gene abundance. B) Resistance gene richness. C) Shannon diversity index. D) Simpson diversity index. E) Chao1 estimator. F) ACE estimator.

Figure S5.

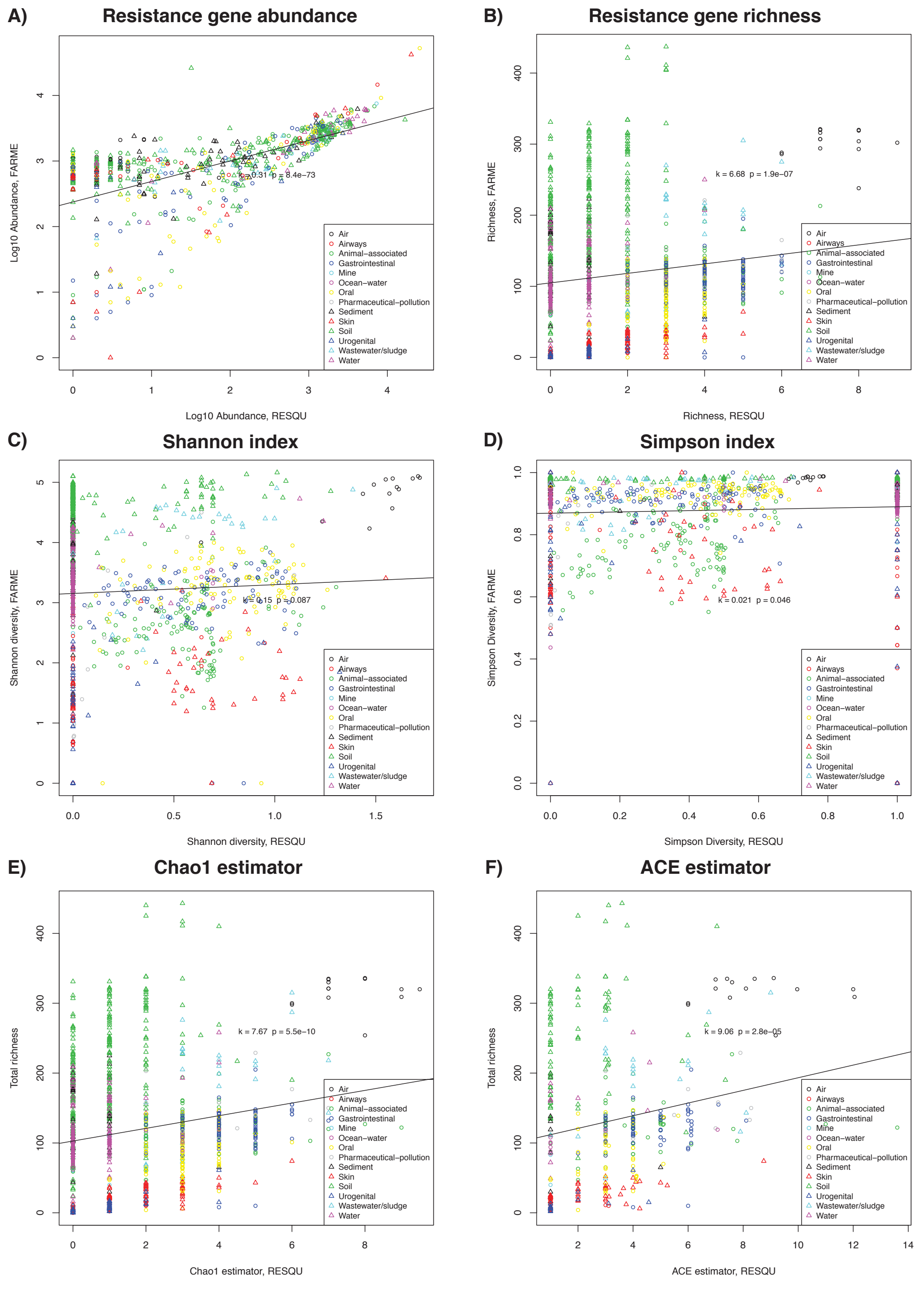

Fig. S5. Relationships between the measurements made based on the ten most predictive resistance genes in Resqu and the total set of FARME genes, in terms of A) total resistance gene abundance, B) resistance gene richness, C) the Shannon diversity index, D) the Simpson diversity index, E) the Chao1 estimator, and F) the ACE estimator.
